# HEY1 functions are regulated by its phosphorylation at Ser-68

**DOI:** 10.1042/BSR20160123

**Published:** 2016-06-03

**Authors:** Irene López-Mateo, Amaia Arruabarrena-Aristorena, Cristina Artaza-Irigaray, Juan A. López, Enrique Calvo, Borja Belandia

**Affiliations:** * Department of Cancer Biology, Instituto de Investigaciones Biomédicas Alberto Sols, CSIC-UAM, Arturo Duperier 4, 28029 Madrid, Spain; † Proteomics Unit, CIC bioGUNE, Bizkaia Technology Park, 48160 Derio, Spain; ‡ División de Inmunología, Centro de Investigación Biomédica de Occidente–IMSS, Sierra Mojada 800, 44340 Guadalajara, Jalisco, Mexico; § Unidad de Proteomica, Centro Nacional de Investigaciones Cardiovasculares, CNIC, 28029 Madrid, Spain

**Keywords:** centrosome, HEY1, p53, phosphorylation, ribosomal stress, STK38

## Abstract

HEY1 (hairy/enhancer-of-split related with YRPW motif 1) is a member of the basic helix–loop–helix-orange (bHLH-O) family of transcription repressors that mediate Notch signalling. HEY1 acts as a positive regulator of the tumour suppressor p53 via still unknown mechanisms. A MALDI-TOF/TOF MS analysis has uncovered a novel HEY1 regulatory phosphorylation event at Ser-68. Strikingly, this single phosphorylation event controls HEY1 stability and function: simulation of HEY1 Ser-68 phosphorylation increases HEY1 protein stability but inhibits its ability to enhance p53 transcriptional activity. Unlike wild-type HEY1, expression of the phosphomimetic mutant HEY1-S68D failed to induce p53-dependent cell cycle arrest and it did not sensitize U2OS cells to p53-activating chemotherapeutic drugs. We have identified two related kinases, STK38 (serine/threonine kinase 38) and STK38L (serine/threonine kinase 38 like), which interact with and phosphorylate HEY1 at Ser-68. HEY1 is phosphorylated at Ser-68 during mitosis and it accumulates in the centrosomes of mitotic cells, suggesting a possible integration of HEY1-dependent signalling in centrosome function. Moreover, HEY1 interacts with a subset of p53-activating ribosomal proteins. Ribosomal stress causes HEY1 relocalization from the nucleoplasm to perinucleolar structures termed nucleolar caps. HEY1 interacts physically with at least one of the ribosomal proteins, RPL11, and both proteins cooperate in the inhibition of MDM2-mediated p53 degradation resulting in a synergistic positive effect on p53 transcriptional activity. HEY1 itself also interacts directly with MDM2 and it is subjected to MDM2-mediated degradation. Simulation of HEY1 Ser-68 phosphorylation prevents its interaction with p53, RPL11 and MDM2 and abolishes HEY1 migration to nucleolar caps upon ribosomal stress. Our findings uncover a novel mechanism for cross-talk between Notch signalling and nucleolar stress.

## Abstract (precis)

HEY1-dependent activation of the p53 tumour suppressor pathway can be inhibited through direct phosphorylation of HEY1 at Ser-68 located in the bHLH domain. STK38 and STK38L serine/threonine kinases can phosphorylate HEY1 Ser-68 and could modulate its biological function.

## INTRODUCTION

Hairy/enhancer-of-split related with YRPW motif proteins are a family of transcription factors that belong to the vertebrate basic-helix–loop–helix-orange (bHLH-O) family of transcriptional repressors [1]. There are three HEY proteins; HEY1, HEY2 and HEYL, which are encoded by three distinct genes. HEY1, like other members of the family, mediates Notch signalling. Upon Notch pathway activation, HEY1 expression increases and it accumulates in the nucleus, leading to transcriptional inhibition of downstream targets. Thus, HEY1 is a critical effector within the Notch signalling pathway during embryonic development [2]. In addition to its roles during embryogenesis, HEY1 has also been linked to several cancer-related pathways. *HEY1* is a direct target gene of transforming growth factor β (TGF-β)/Smad signalling and HEY1 expression is essential for TGF-β-dependent epithelial-to-mesenchymal transition, a developmental program of cell plasticity frequently observed in advanced carcinogenesis [3]. The retinoblastoma (pRb)/E2F cell-cycle pathway can up-regulate *HEY1* expression in human glioma cells through E2F-binding sites present in its promoter [4], and HEY1 expression can also be induced by activation of the proto-oncogene c-Jun [5]. HEY1 has recently gained relevance to cancer because it has been shown that it is a positive regulator of the p53 tumour suppressor protein (TP53 or p53), a transcription factor key in cancer protection that regulates the expression of stress response genes, which in turn prevent damaged cells to initiate malignant growth. Therefore, *TP53* is the most frequently mutated tumour suppressor in human cancers [6]. HEY1 expression activates p53 and induces apoptosis *in vivo* in different biological models and it was proposed that these effects occur through transcriptional repression of MDM2, a p53-specific E3 ubiquitin ligase that targets p53 to proteasome for degradation [7]. In addition, HEY1 expression results in p53-dependent growth arrest in Ewing sarcoma family cancer cell lines [8]. Lastly, our laboratory demonstrated that HEY1-dependent activation of p53 blocks cell proliferation in human osteosarcoma cells (U2OS) and confers sensitivity to p53-activating cancer drugs [9]. Therefore, there is a clear contribution of HEY1 to the activation of p53, which elicits different biological responses depending on the cellular context, although little is known about the molecular mechanisms that underlie this functional interaction. Moreover, alterations in the normal function of HEY1-dependent pathways could affect p53 tumour suppression function, contributing to cancer development.

To further understand the role of HEY1 in p53 signalling we carried out a proteomic approach combining immunoprecipitation with liquid chromatography coupled to tandem mass spectrometry, designed to uncover the functional interactions of HEY1 with cellular proteins and the post-translational modifications present in those proteins. Here we describe a critical regulatory phosphorylation event at HEY1 Ser-68 residue that modulates its function as activator of p53 transcriptional activity. Detailed studies carried out with phosphomimetic aspartic acid substitutions or unphosphorylatable alanine substitutions at HEY1 Ser-68 residue revealed the effects that simulation of HEY1 Ser-68 phosphorylation have in its stability and function. In addition we have identified two related kinases, STK38 (NDR1) and STK38L (NDR2), which interact with and phosphorylate HEY1 at Ser-68 residue and could have a previously unknown role in the regulation of its function. We also present evidence for a possible novel role of HEY1 in the mediation and/or modulation of the ribosomal protein (RP)/MDM2/p53 axis, responsible for the activation of p53 upon nucleolar stress.

## MATERIALS AND METHODS

### Plasmids

The following plasmids have been described: pSG5-HEY1, GST-HEY1 (full-length and deletion mutants ΔY, amino acids 1–285; ΔY+O, amino acids 1–115; ΔY+O+H, amino acids 1–49; ΔHLH, amino acids 116–299), pSG5-HEY2 [10], PIG3-LUC and pCDNA-p53 [9], pSG5-HEYL [11], pCMV-MDM2 [12]. The complete open reading frame of human RPL11 was amplified by PCR from pcDNA-myc3-L11 [13] and subcloned into pSG5-Flag [10]. The complete open reading frame of human NONO was amplified by PCR from cDNA obtained from human U2OS cells and subcloned into pSG5-Flag. pSG5-HEY1-S68D, pSG5-HEY1-S68A, pSG5-HEY1-S246D, pSG5-HEY1-S246A, GST-HEY1-S68D and GST-HEY1-S68A were generated by PCR site-directed mutagenesis.

### Cell culture and transient transfections

U2OS [14] and H1299 [15] cells were cultured in Dulbecco's modified Eagle's medium supplemented with 10% fetal bovine serum. Both cell lines were generous gift from Dr Susana Llanos (Spanish National Cancer Research Center, CNIO). Twenty-four hours before transfection, cells were plated in 24-well plates (50000 cells per well), 60-mm dishes (500000 cells per dish) or 150-mm dishes (3500000 cells per dish). Cells were transfected using Lipofectamine LTX (Life Technologies). Transfected plasmids are detailed in the figure legends. pRL-TK (10 ng/well, Promega) was used as internal control for transfection efficiency in luciferase assays. Cell extracts were assayed for luciferase activity as described previously [10] or lysed to obtain whole cell extracts.

### Generation of U2OS polyclonal cell pools expressing tetracycline-inducible HEY1 or HEY1-S68D.

Lentiviral vectors encoding V5-tagged HEY1 or HEY1-S68D were used to generate lentivirus expressing HEY1 or HEY1-S68D by the ViraPower™ T-REx™ system following manufacturer's instructions (Invitrogen). Cells were co-transduced with Tet-repressor-lentivirus and either HEY1- or HEY1-S68D-lentivirus. Selection of stably co-transduced cells was achieved with Zeocin (1000 μg/ml) and Blasticidin (4 μg/ml). To induce HEY1 or HEY1-S68D expression 1 μg/ml of tetracycline was added to the media.

### Proliferation assays

U2OS cells expressing tetracycline-inducible HEY1 or HEY1-S68D were plated into 96-well plates (2500 cells/well). After 24 h 1 μg/ml of tetracycline was added and cell proliferation was measured at different time points. Medium and tetracycline were replaced every 3 days. Cell growth was determined in quadruplicates using the CellTiter One-Solution-Assay (Promega) and reading absorbance at 490 nm.

### GST pull-down assays

Expression vectors were transfected into U2OS cells using Lipofectamine LTX, GST fusion proteins were induced, purified, bound to Sepharose beads (GE Healthcare), and incubated with U2OS whole cell extracts as described previously [16] in NETN buffer (20 mM Tris/HCl (pH 8.0), 1 mM EDTA, 0.5% Nonidet P-40, 100 mM NaCl). After extensive washing, the samples were separated on SDS/10% polyacrylamide gels. Gels were blotted on to nitrocellulose and probed with antibodies. When comparing interactions all GST-fusion proteins were diluted at similar concentrations.

### Antibodies and immunoblotting

The antibodies used were anti-Flag M2 (F1804, Sigma–Aldrich, dilution 1:1000), anti-p53 (DO-1, sc-126, Santa Cruz Biotechnology, dilution 1:1000), anti-MDM2 (SMP14, sc-965, Santa Cruz Biotechnology, dilution 1:1000), anti-γ-tubulin (C-11, sc-17787, Santa Cruz Biotechnology) and anti-β-actin (AC-15, sc-69879, Santa Cruz Biotechnology, dilution 1:10000). Phospho-HEY1 (Ser-68) rabbit polyclonal antibody was generated using the phosphorylated peptide DRINN(pS68)LSELRRL-cys as immunogen (Abyntek Biopharma S.L.). The antibodies were cross-adsorbed with a non-phosphopeptide column to remove antibodies against the non-phospho-specific epitope. Antibody specificity was verified by ELISA and DOT BLOT (Supplementary Figure S1).

### Immunofluorescence analysis

U2OS grown on coverslips were transfected with 100 ng of the plasmids indicated in the figure legends. After 24 h, cells were fixed with 4% paraformaldehyde in PBS for 15 min and permeabilized in 0.1% Triton X-100 in PBS for 5 min. To perform indirect immunofluorescence cells were incubated in 3% BSA in PBS for 30 min. Primary immunostaining with mouse anti-Flag antibody (1:500) or mouse anti-γ-tubulin (1:100) was carried out at RT for 1 h. Secondary immunostaining with Alexa Fluor 488 goat anti-mouse antibody (A11029, Life Technologies) was performed at RT for 1 h. DNA was counterstained with DAPI. Stained cells were mounted on glass slides and examined using an Eclipse 90i microscope (Nikon).

### Immunoprecipitation

U2OS cells, previously transfected with Flag-HEY1, were lysed by incubation for 20 min at 4°C, in NETN buffer (20 mM Tris/HCl (pH 8.0), 1 mM EDTA, 0.5% Nonidet P-40, 100 mM NaCl). After centrifugation at 14000 ***g*** for 15 min at 4°C, the supernatants were used for immunoprecipitation with anti-Flag M2 magnetic beads (Sigma–Aldrich), previously washed four times in the same NETN buffer, at 4°C for 90 min. The immune complexes with the magnetic beads were initially washed three times with the NETN buffer. To perform liquid chromatography coupled to tandem mass spectrometry the immune complexes were washed five more times with the NETN buffer without Nonidet P-40 and five times with 50 mM ammonium bicarbonate. The magnetic beads were finally resuspended in ammonium bicarbonate and subject to proteomic analysis.

### Proteomic analysis by liquid chromatography coupled to tandem mass spectrometry

Samples were digested by adding modified porcine trypsin (Promega) at a final ratio of 1:50 (trypsin–protein). Digestion proceeded overnight at 37°C. After digestion, samples were vacuum-dried and finally dissolved in 1% acetic acid for liquid chromatography coupled to tandem mass spectrometry analysis as described previously [17].

## RESULTS

### Simulation of HEY1 phosphorylation at residue S68 inhibits its ability to enhance p53 transcriptional activity

**Figure 1:**
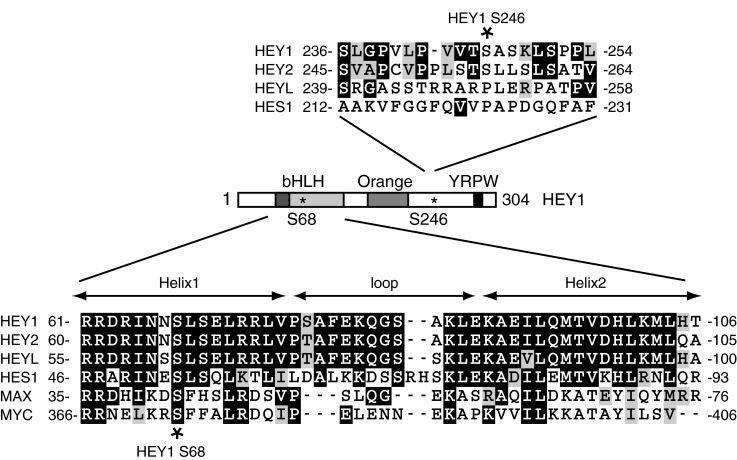
Schematic representation of HEY1 The asterisks indicate the two phosphorylated serine residues identified in the proteomic study (Ser-68 and Ser-246). The helix–loop–helix domain of HEY1, HEY2, HEYL, HES1, MYC and MYC-associated factor (MAX), and the region around HEY1 Ser-246 and the equivalent serine in HEY2, HEYL and HES1 were aligned by ClustalW2 and formatted with BOXSHADE. Identical amino acids are in black, and conserved residues are in grey.

**Figure 2:**
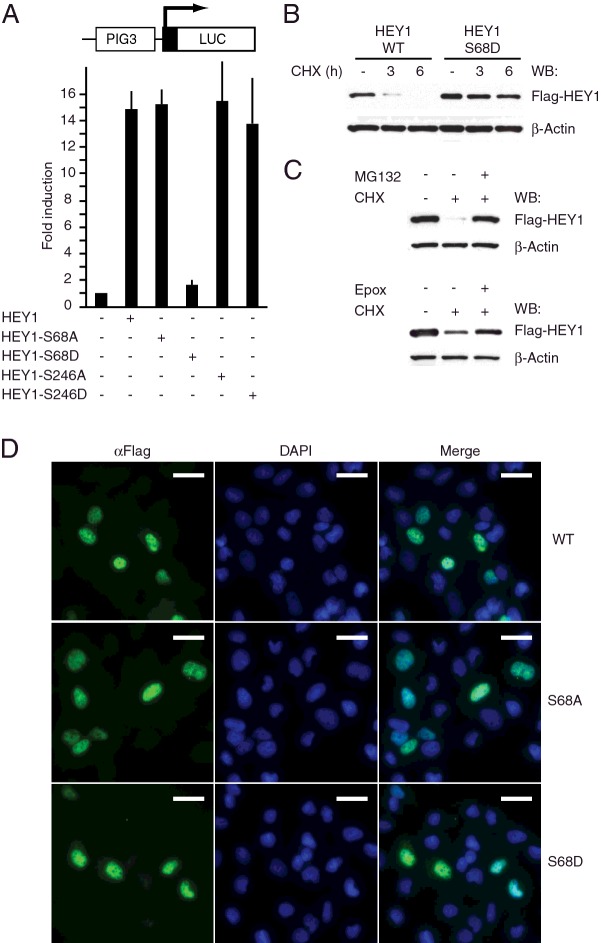
Simulation of HEY1 phosphorylation at residue Ser-68 inhibits its ability to enhance p53 transcriptional activity (**A**) U2OS cells were transfected with 100 ng of PIG3-LUC and 200 ng of expression vectors for HEY1, HEY1-S68A, HEY1-S68D, HEY1-S246A or HEY1-S246D. After transfection, cells were incubated 24 h. Subsequently cell lysates were assayed using a dual luciferase reporter system. Normalized values are expressed relative to the activity of the reporter in the absence of HEY1. The results shown represent the averages of results of three independent experiments assayed in duplicate + S.D. (**B**) Simulation of HEY1 phosphorylation at residue S68 increases protein stability. U2OS cells were transfected with expression vectors for Flag-tagged HEY1 or HEY1-S68D. Twenty-four hours after transfection cells were treated with cycloheximide (CHX, 10 μg/ml) and HEY1 protein levels were analysed by western blotting at 0, 3 and 6 h after CHX addition. (**C**) HEY1 is degraded via proteasome. Degradation of HEY1 protein following cycloheximide treatment was prevented by addition of different proteasome inhibitors; MG132 (25 μM) and Epoxomicin (Epox, 1 μM). Anti-β-actin antibody was used as a loading control for all western blots. (**D**) Simulation of HEY1 phosphorylation does not affect HEY1 nuclear localization. U2OS cells were transfected with expression vectors for Flag-tagged HEY1, HEY1-S68A or HEY-S68D and assayed by indirect immunofluorescence with anti-Flag antibody. The first column shows the indirect immunofluorescence with anti-Flag antibody, the second column shows DAPI staining of DNA and the third column shows the merge image indicating the degree of colocalization. Bars, 20 μm.

To identify proteins that interact with HEY1, and possible post-translational modifications that occur in those proteins, we transfected Flag-tagged HEY1 expression vector into U2OS cells and we performed immunoprecipitation with anti-Flag magnetic beads. The immunocomplexes were analysed by using a proteomic approach based on liquid chromatography coupled to tandem mass spectrometry (LC–MS/MS). The present study identified two serine residues phosphorylated in HEY1, Ser-68 and Ser-246. Ser-68 is located in the middle of helix 1 within the HLH domain, and is highly conserved between members of the bHLH-O family and less closely related bHLH transcription factors such as MYC and MAX (Figure 1). Ser-246 is located in a region of HEY1 currently without known function and is only conserved in the closely related HEY2 protein (Figure 1). Little is known about the molecular mechanisms that regulate HEY1 function so we investigated a possible regulatory role for those phosphorylation events in HEY1 biological activity. We generated HEY1 mutants in which Ser-68 or Ser-246 were replaced by aspartic acid or alanine in an attempt to mimic or block their phosphorylation respectively. We tested the effects of those mutations in the ability of HEY1 to stimulate transcription from p53-responsive luciferase genes in transfected cells. Interestingly, the phosphomimetic substitution S68D almost completely abolished the ability of HEY to stimulate p53 transcriptional activity, whereas the rest of mutations did not significantly affect HEY1 activity (Figure 2A, Supplementary Figure S2). Western blot analysis showed that the lack of function does not reflect lower protein expression because HEY1-S68D steady-state protein levels are higher than wild-type HEY1 (Figure 2B). This result reflects an increase in HEY1 protein stability caused by the mutation S68D, as demonstrated by comparing the levels of wild-type HEY1 and HEY1-S68D in U2OS cells after treatment with the protein synthesis inhibitor cycloheximide (Figure 2B). Experiments performed with MG-132, an inhibitor of proteasome and calpains, suggested that HEY1 is degraded by the ubiquitin-proteasome pathway ([18] and Figure 2C, top panel). Addition of the more proteasome-specific inhibitor epoxomicin confirmed that HEY1 is mainly degraded by the proteasome (Figure 2C, bottom panel). HEY1 is a nuclear protein, however nuclear exclusion of HEY1 has been observed in prostate cancer cells [10] and this alteration prevents HEY1-dependent p53 activation [9]. To study whether simulation of HEY1 phosphorylation at Ser-68 affects its cellular localization we performed immunofluorescence analysis. Thus, we observed that none of the phosphorylation site mutants alter the normal nuclear localization of HEY1 (Figure 2D).

### Simulation of HEY1 phosphorylation at residue S68 specifically alters HEY1 protein–protein interactions *in vitro*

**Figure 3:**
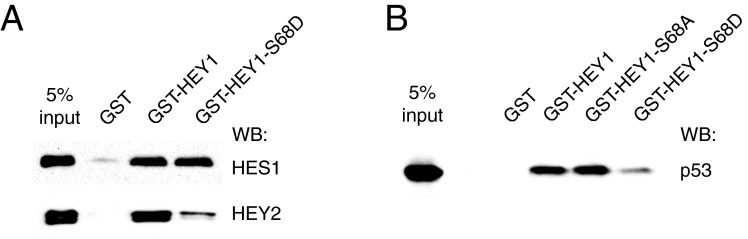
Simulation of HEY1 phosphorylation at residue Ser-68 specifically alters HEY1 protein–protein interactions (**A**) Whole-cell extracts from U2OS cells previously transfected with expression vectors for Flag-tagged HES1 or HEY2 were incubated with GST fusion proteins of HEY1 or HEY1-S68D coupled with Sepharose beads. The associated proteins were detected by western blotting using anti-Flag antibody. (**B**) Whole-cell extracts from U2OS cells previously transfected with expression vector for p53 were incubated with GST fusion proteins of HEY1, the mutant HEY1-S68A, or HEY1-S68D coupled with Sepharose beads. The associated proteins were detected by western blotting using anti-p53 antibody.

HEY1 forms functional hetero and homo-dimers with other members of the bHLH-O superfamily. The Ser-68 is located in the Helix 1, within the HLH domain, the main determinant of the homo- or hetero-dimerization surface between bHLH proteins [19]. To address whether Ser-68 phosphorylation state affect the interaction between HEY1 and other bHLH proteins, we performed GST pull-down experiments. GST-wild-type-HEY1 and GST-HEY1-S68D interacted in a similar manner with HES1 (hairy and enhancer of split 1), however simulation of Ser-68 phosphorylation greatly reduced the ability of HEY1 to interact with HEY2 (Figure 3A), suggesting that this phosphorylation event modulates specifically HEY1 protein-protein interactions with other bHLH-O proteins. HEY1 also interacts directly with p53 *in vitro* [20] and the mutant HEY1-S68D showed reduced ability to interact with p53 as compared with wild-type HEY1 (Figure 3B). The reduced interaction does not reflect lower GST-HEY1-S68D expression because control Coomassie blue-stained gels shown that it expresses at even higher levels than GST-wild-type-HEY1 (Supplementary Figure S3), and the input of GST fusion proteins used in the experiments were similar. Together, these data indicate that the phosphorylation status of HEY1 Ser-68 modulates specifically HEY1 protein–protein interactions.

### HEY2 function is also regulated through phosphorylation at the conserved serine in the HLH domain

**Figure 4:**
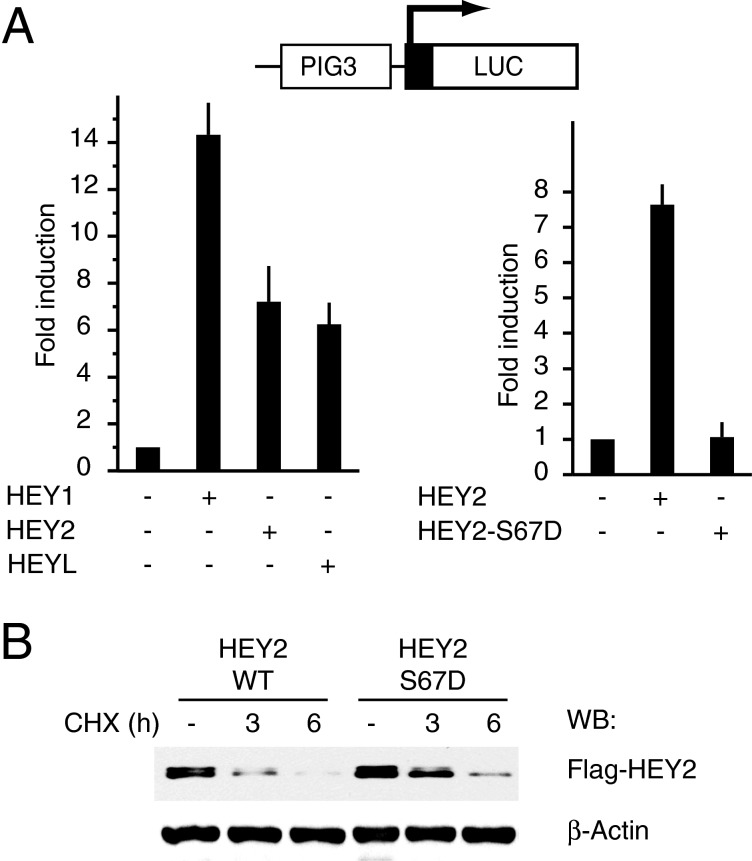
Simulation of HEY2 phosphorylation at residue Ser-67 inhibits its ability to enhance p53 transcriptional activity (**A**) U2OS cells were transfected with 100 ng of PIG3-LUC and 200 ng of expression vectors for HEY1, HEY2 or HEYL (left panel) or expression vectors for HEY2 or HEY2-S67D (right panel). After transfection, cells were incubated 24 h. Subsequently cell lysates were assayed using a dual luciferase reporter system. Normalized values are expressed relative to the activity of the reporter in the absence of HEY1. The results shown represent the averages of results of three independent experiments assayed in duplicate + S.D. (**B**) Simulation of HEY2 phosphorylation at residue Ser-67 increases protein stability. U2OS cells were transfected with expression vectors for Flag-tagged HEY2 or HEY2-S67D. Twenty-four hours after transfection cells were treated with cycloheximide (CHX, 10 μg/ml) and HEY2 protein levels were analysed by western blotting at 0, 3 and 6 h after CHX addition.

The conservation of a serine residue equivalent to HEY1 Ser-68 among all bHLH-O proteins suggests that its phosphorylation could be a common regulatory mechanism. To investigate this possibility we generated a HEY2-S67D phosphomimetic mutant at the equivalent Ser-67 residue in HEY2. First we confirmed that all three members of HEY family, HEY1, HEY2 and HEYL, are able to stimulate p53 transcriptional activity in transient transfection experiments (Figure 4A, left panel). Subsequently, we compared the p53-activating potential of wild-type HEY2 with the mutant HEY2-S67D and we observed that simulation of HEY2 Ser-67 phosphorylation abolished its ability to stimulate p53 transcriptional activity (Figure 4A, right panel). Moreover, in a similar fashion than HEY1, the mutant HEY2-S67D protein is more stable than wild-type HEY2 (Figure 4B). Hence, it appears that this phosphorylation could be a general regulatory mechanism for all members of the bHLH-O family.

### Simulation of HEY1 phosphorylation at residue S68 inhibits HEY1 ability to induce p53-dependent cell cycle arrest

**Figure 5:**
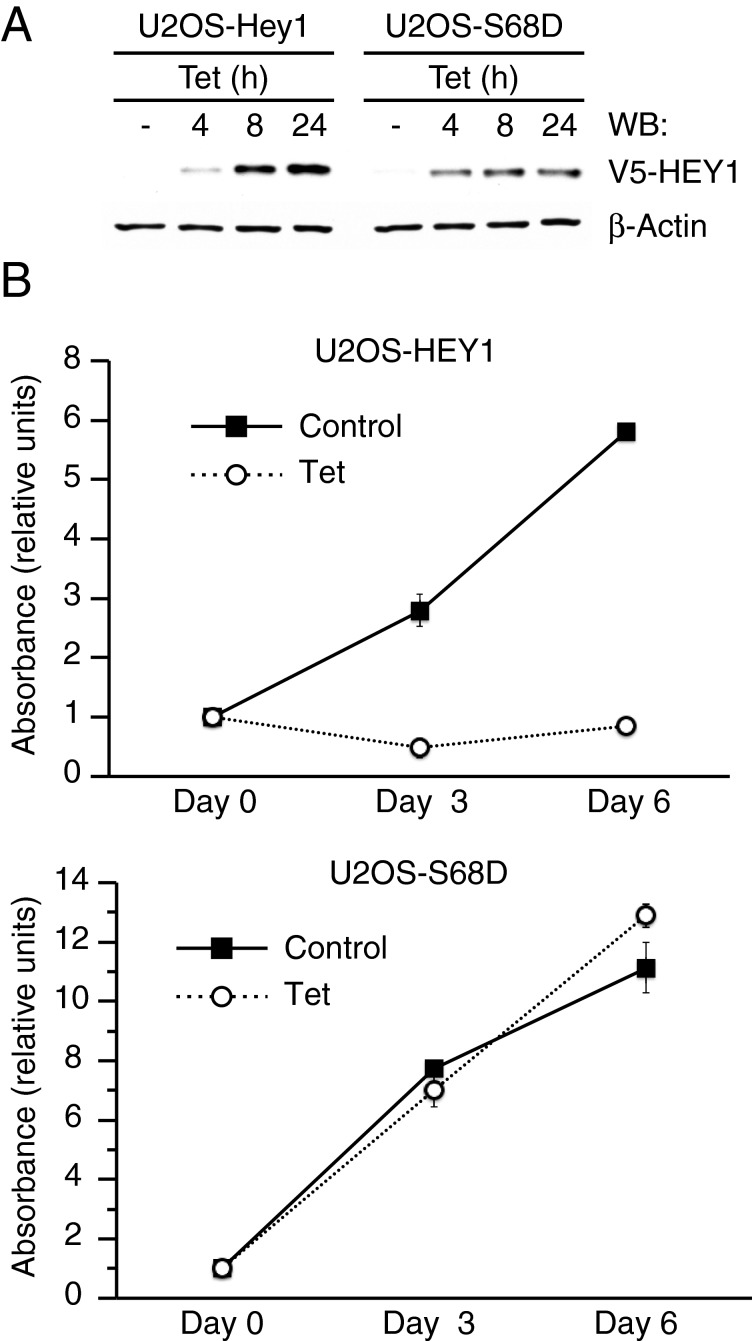
Simulation of HEY1 phosphorylation at residue Ser-68 inhibits HEY1 ability to induce p53-dependent cell cycle arrest (**A**) Immunoblot analysis of HEY1 expression in stable U2OS cell lines expressing inducible HEY1 (U2OS-HEY1) or the phosphomimetic mutant HEY1-S68D (U2OS-S68D) treated with 1 μg/ml tetracycline (Tet) for 4, 8 or 24 h. The protein levels of β-actin are shown as a loading control. (**B**) U2OS-HEY1 cells (top panel) or U2OS-S68D cells (bottom panel) were treated either with vehicle (Control) or 1 μg/ml tetracycline (Tet). Cell proliferation was monitored at different time points by using 3-(4,5-dimethylthiazol-2-yl)-5-(3-carboxymethoxyphenyl)-2-(4-sulfophenyl)-2H-tetrazolium (MTS) assay. The results shown represent the averages of results of two independent experiments assayed in quadruplicate ± S.D.

HEY1 expression induces p53-dependent growth arrest in Ewing's sarcoma family tumour cells [8] and we have previously shown that HEY1 expression induces p53-dependent cell-cycle arrest and aberrant cell differentiation in human osteosarcoma U2OS cells [9]. To examine whether simulation of Ser-68 phosphorylation affects HEY1 function we generated tetracycline-inducible pools of U2OS cells stably expressing either HEY1 (U2OS-HEY1) or HEY1-S68D (U2OS-S68D) (Figure 5A). As expected, induction of wild-type HEY1 expression inhibited cell proliferation (Figure 5B). In contrast, induction of HEY1-S68D expression did not stop cell proliferation (Figure 5B). HEY1 effects on cell proliferation are accompanied by neuron-like differentiation, down-regulation of expression of components of the Notch pathway and RUNX2, a master regulator of osteoblast differentiation, and modulation of expression of several cell-cycle regulatory genes [9]. However, expression of the phosphomimetic mutant HEY1-S68D failed to reproduce these effects (Supplementary Figure S4). These results suggest that HEY1 phosphorylation at residue Ser-68 could play an important role in the regulation of HEY1 functions *in vivo*.

### Expression of the phosphomimetic mutant HEY1-S68D failed to sensitize U2OS cells to p53-activating chemotherapeutic drugs

**Figure 6:**
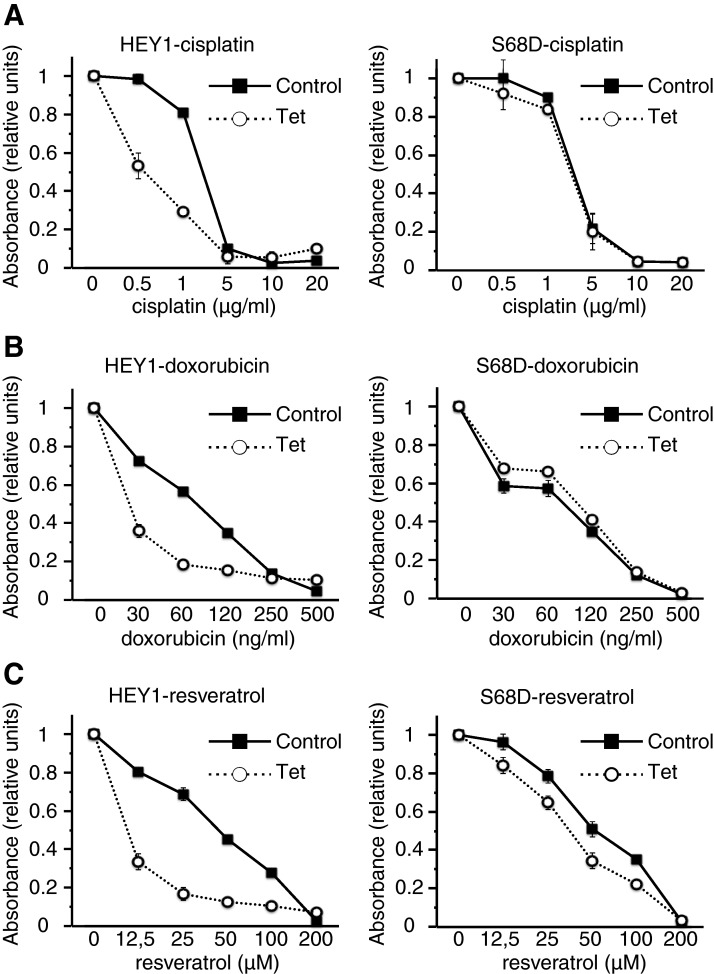
Expression of HEY1 but not HEY1-S68D leads to increased sensitivity to cisplatin, doxorubicin and resveratrol U2OS-HEY1 cells (left panels) or U2OS-S68D cells (right panels) were treated with 1 μg/ml tetracycline (Tet) to induce expression of HEY1 or HEY1-S68D or with vehicle (Control). After 8 h cells were cultured with varying concentrations of cisplatin (**A**), doxorubicin (**B**) or resveratrol (**C**) for another 72 h in the presence and absence of tetracycline. Subsequently, cell viability was assayed using an MTS-based assay. Data were plotted relative to the drug-free controls. The results shown represent the averages of results of two independent experiments assayed in quadruplicate ± S.D.

p53 plays a central role in the sensitivity to chemotherapy [21] and we previously shown that expression of HEY1 confers sensitivity to p53-activating chemotherapeutic drugs on U2OS cells [9]. To determine whether simulation of Ser-68 phosphorylation also modulates HEY1-dependent sensitization to chemotherapeutic agents we performed cytotoxicity assays using cisplatin and doxorubicin. As previously reported, induction of HEY1 expression in U2OS-HEY1 cells resulted in an increase in sensitivity to both drugs (Figures 6A and 6B). However, induction of HEY1-S68D expression in U2OS-S68D cells did not alter the sensitivity of U2OS cells to any of the drugs (Figures 6A and 6B), in accordance with the above describe failure of this mutant to activate p53-dependent signalling. We extended our study to possible effects of HEY1 expression on cellular sensitivity to resveratrol, a naturally occurring polyphenol that appears to have many anti-tumour effects on different cancer cells [22] mediated, at least in part, by activation of p53 [23]. Again, as in the case of cisplatin and doxorubicin, we observed that expression of HEY1, but not of the mutant HEY1-S68D, conferred sensitivity to resveratrol on U2OS cells (Figure 6C). Thus, it seems that HEY1 expression causes a general sensitization of U2OS cells to anticancer drugs, an effect that might be regulated by HEY1 Ser-68 phosphorylation.

### STK38 and STK38L kinases phosphorylate HEY1 *in vitro*

**Figure 7:**
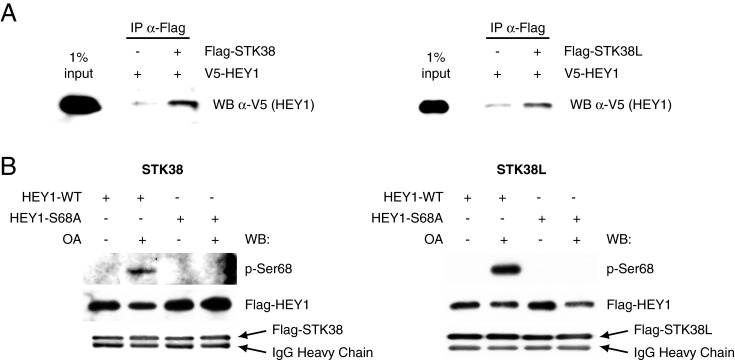
STK38 and STK38L kinases phosphorylate HEY1 *in vitro* (**A**) *In vitro* interaction of STK38 kinases with HEY1. Whole-cell extracts from U2OS cells previously transfected with expression vectors for V5-tagged HEY1 were incubated with Flag-tagged-STK38 or STK38L coupled with anti-Flag magnetic beads. The associated proteins were detected by western blotting using anti-V5 antibody. (**B**) HEY1 is phosphorylated at serine 68 *in vitro* by okadaic acid-activated STK38 and STK38L kinases. U2OS cells expressing Flag-tagged STK38 or STK38L kinases were incubated for 1 h with vehicle or 1 μM okadaic acid. Flag-tagged kinases were then immunoprecipitated and assayed for kinase activity using immunopurified FLAG-tagged wild-type HEY1 or the non-phosphorylatable HEY1-S68A mutant as substrate. Phosphorylation of HEY1 was determined by western blotting with anti-phospho-S68 antibody. Western blotting with anti-Flag antibody shows the amount of Flag-HEY1, Flag-STK38 or Flag-STK38L present in each assay.

Among the proteins that co-immunoprecipitated with HEY1 in our proteomic study there were only four serine/threonine kinases (Supplementary Table S1) and, by far, the two closely related STK38 (serine/threonine kinase 38) and STK38L (serine/threonine kinase 38 like) kinases (also known as NDR1 and NDR2 respectively) were the most frequently represented protein kinases. We confirmed that HEY1 was able to interact *in vitro* with STK38 and STK38L by using immunoprecipitation assays (Figure 7A). These observations prompted us to investigate whether these two kinases could phosphorylate HEY1 Ser-68. Both STK38 and STK38L kinases are potently activated upon treatment of intact cells with okadaic acid due to the inhibition of protein phosphatase 2A [24]. We transfected Flag-tagged STK38 and STK38L into U2OS cells and purified the kinases from cells treated (activated kinases) or untreated (inactive kinases) with okadaic acid. These kinases were then used in an *in vitro* kinase assay using affinity purified Flag-HEY1 or the nonphosphorylatable Flag-HEY1-S68A as a substrate. To detect the possible phosphorylation of HEY1 by STK38 kinases we generated an antibody that detects phosphorylation at Ser-68. Thus, we observed in western blot assays that both STK38 and STK38L, when activated upon okadaic acid treatment, were able to phosphorylate wild-type HEY1 at Ser-68 (Figure 7B).

### HEY1 phosphorylated at Ser-68 localizes at the centrosome of mitotic but not interphase U2OS-HEY1 cells

**Figure 8:**
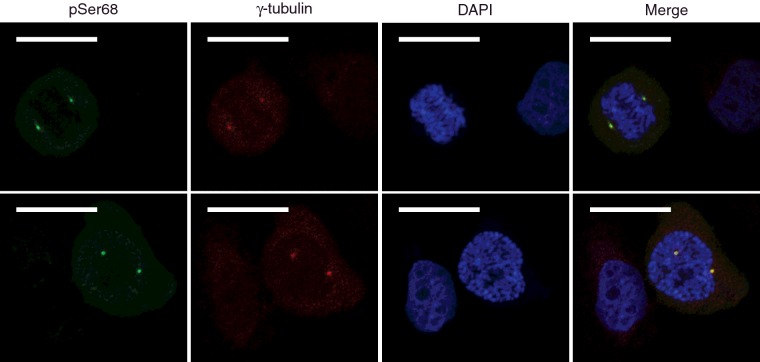
Confocal section of U2OS-HEY1 cells showing high concentration of HEY1 phosphorylated at Ser-68 in the centrosomes of mitotic cells U2OS-HEY1 cells were treated with 1 μg/ml tetracycline to induce expression of HEY1. After 6 h cells were fixed and processed for double immunofluorescence staining using antibodies specific for HEY1-phospho-S68 and the centrosome marker γ-tubulin. DAPI is used to counterstain the nucleus. Bars, 20 μm. Two representative confocal microscopy images are shown.

HEY1 is a nuclear protein [25] and we observed that both HEY1 Ser-68 mutants (S68A and S68D), similar to wild-type HEY1, maintained uniform nuclear expression (Figure 2D). To investigate whether HEY1 phosphorylation at Ser-68 could occur *in vivo* at discrete nuclear structures, the localization of HEY1 phosphorylated at Ser-68 was assessed by confocal indirect immunofluorescence. U2OS-HEY1 cells were incubated with tetracycline for 6 h to induce HEY1 expression. Cells were then immunostained using the anti-phospho-Ser-68-specific antibody. We found that HEY1 phosphorylated at Ser-68 accumulate in two discrete spots observed only in mitotic cells (ascertained by mitotic chromatin condensation), in a number and arrangement of spots resembling that of centrosomes. Double immunofluorescence experiments using anti-phospho-Ser-68-specific antibody in combination with antibody against the centrosomal marker γ-tubulin showed that HEY1 phosphorylated at Ser-68 localizes at centrosomes during mitosis (Figure 8). A functional relationship between HEY1 and the centrosome is reinforced by the high ratio of centrosomal proteins, or centrosome-associated proteins, that co-immunoprecipitated with HEY1 in the proteomic study (47 out 278 total proteins, Supplementary Table S2). Collectively, these observations suggest that HEY1 could play a role in the regulation of centrosome function and this functional interaction could be regulated by HEY1 phosphorylation at Ser-68 in a cell cycle-dependent manner.

### HEY1 interacts with multiple ribosomal proteins

**Figure 9:**
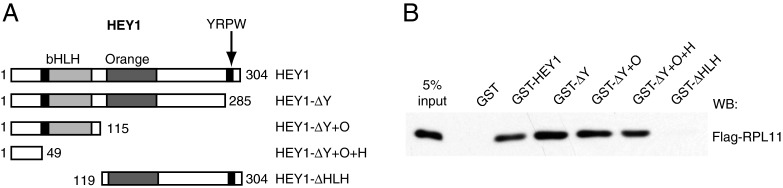
*In vitro* interaction of RPL11 with HEY1 Whole cell extracts from U2OS cells previously transfected with expression vector for Flag-tagged RPL11 were incubated with GST fusion proteins of full-length HEY1 or deletion mutants (**A**) coupled with Sepharose beads. Bound proteins were detected by western blotting using anti-Flag antibody (**B**).

Gene Ontology analysis of the genes encoding the proteins identified in the proteomic study (performed using STRING Web resources, [26]) showed that among the top most enriched groups were genes encoding proteins involved in ribosome assembly and/or function (Supplementary Table S3). During the last few years, perturbation of ribosomal biogenesis has emerged as an important p53-regulatory pathway [27], and our study revealed that many ribosomal proteins potentially interact with HEY1 (Supplementary Table S4). Moreover, seven ribosomal proteins directly involved in the regulation of p53 activity co-precipitated with HEY1; RPS7, RPL23, RPL11, RPS20, RPS25, RPS14 and RPS3 [27–30]. A GST pull-down assay demonstrated that at least one of them, RPL11, interacts directly with HEY1 (Figure 9B). Using various HEY1 deletion mutants fused to GST we mapped the RPL11-interacting domain of HEY1 to a small N-terminal region (amino acids 1–49) of previously unknown function (Figure 9).

### HEY1 and RPL11 cooperate to inhibit MDM2-mediated p53 degradation

**Figure 10:**
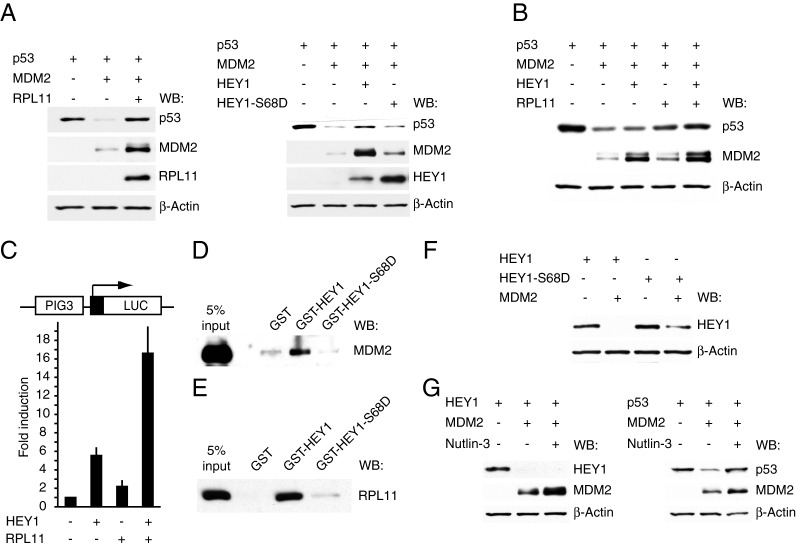
Functional interaction between HEY1 and RPL11 (**A**) Wild-type HEY1, but not the mutant HEY1-S68D, prevents MDM2-mediated p53 degradation. H1229 cells were transfected, where indicated, with expression vectors for p53 (10 ng), MDM2 (1 μg), RPL11 (1 μg), HEY1 (1 μg) or HEY1-S68D (1 μg). Twenty-four hours after transfection cells were harvested for immunoblot analysis with the specified antibodies. (**B**) RPL11 cooperates with HEY1 to prevent MDM2-mediated p53 degradation. H1229 cells were transfected, where indicated, with expression vectors for p53 (10 ng), MDM2 (1 μg), RPL11 (0.5 μg) or HEY1 (0.5 μg). Twenty-four hours after transfection cells were harvested for immunoblot analysis with the specified antibodies. (**C**) RPL11 cooperates synergistically with HEY1 to enhance p53 transcriptional activity. U2OS cells were transfected with 100 ng of PIG3-Luc in the presence or absence of 100 ng of expression vector for HEY1, 50 ng of expression vector for RPL11 or both together. After transfection, cells were incubated 24 h. Subsequently cell lysates were assayed using a dual luciferase reporter system. Normalized values are expressed relative to the activity of the reporter in the absence of HEY1. The results shown represent the averages of results of four independent experiments assayed in duplicate + S.D. (**D**) Simulation of HEY1 phosphorylation at residue S68 inhibits its interaction with MDM2. Whole-cell extracts from U2OS cells previously transfected with expression vector for MDM2 were incubated with GST fusion proteins of HEY1 or HEY1-S68D coupled with Sepharose beads. The associated proteins were detected by western blotting using anti-MDM2 antibody. (**E**) Simulation of HEY1 phosphorylation at residue S68 inhibits its interaction with RPL11. Whole-cell extracts from U2OS cells previously transfected with expression vector for Flag-tagged RPL11 were incubated with GST fusion proteins of HEY1 or HEY1-S68D coupled with Sepharose beads. The associated proteins were detected by western blotting using anti-Flag antibody. (**F**) MDM2 decreases the level of ectopically expressed HEY1. U2OS cells were transfected with expression vectors for MDM2 (2 μg) and Flag-tagged HEY1 (2 μg) or HEY1-S68D (2 μg). Twenty-four hours after transfection cells were harvested for immunoblot analysis with the specified antibodies. (**G**) Effects of Nutlin-3 on MDM2-mediated degradation of HEY1 or p53. H1229 cells were transfected, where indicated, with expression vectors for HEY1 (1 μg), p53 (10 ng) and MDM2 (1 μg). Eighteen hours after transfection cells were washed and incubated with vehicle or 10 μM Nutlin-3 for an additional 24 h. Subsequently, cells were harvested for immunoblot analysis with the specified antibodies.

RPL11, like other ribosomal proteins, activates p53 by binding to MDM2 and suppressing its E3 ubiquitin ligase activity in response to ribosomal stress [31]. Thus, RPL11 expression protects against MDM2-directed p53 degradation when co-transfected in p53-null human lung adenocarcinoma H1299 cells (Figure 10A, left panel). Remarkably, in a similar experiment, wild-type HEY1 expression, but not HEY1-S68D expression, was also able to prevent MDM2-mediated p53 degradation (Figure 10A, right panel). Expression of wild-type HEY1 also promotes MDM2 accumulation, possibly through inhibition of MDM2 self-ubiquitination and degradation, as previously shown for RPL11 ([31], Figure 10A). These results suggest that HEY1 activates p53 signalling, at least in part, through inhibition of MDM2 ubiquitin ligase activity. Moreover, this previously unknown function of HEY1 can be regulated by phosphorylation of Ser-68. Parallel experiments performed in H1299 cells transfected with suboptimal amounts of HEY1 and RPL11 expression plasmids allowed us to observe that HEY1 and RPL11 cooperate in preventing MDM2-mediated p53 degradation (Figure 10B). To further analyse the cooperation between HEY1 and RPL11 in p53 activation we co-transfected suboptimal amounts of HEY1 and RPL11 expression plasmids, either independently or in combination, together with the p53-dependent PIG3-LUC reporter. We observed a synergistic activation of PIG3-LUC reporter activity (Figure 10C), reinforcing the idea that both proteins may cooperate in the activation of p53.

Many ribosomal proteins activate p53 by direct binding to MDM2. To explore the possibility that HEY1 could also interact with MDM2 we performed a pull-down interaction assay. This showed that GST-HEY1 was able to interact weakly but specifically with MDM2 (Figure 10D). Interestingly, simulation of HEY1 Ser-68 phosphorylation completely abolished this interaction. Furthermore, simulation of Ser-68 phosphorylation also inhibits the *in vitro* interaction between RPL11 and HEY1 (Figure 10E). These results indicate that there is a direct correlation between HEY1 ability to interact with RPL11 and MDM2 and its p53-activating activity, and these functional interactions could be regulated by HEY1 phosphorylation at Ser-68.

The physical interaction between HEY1 and MDM2 suggests that HEY1 could be substrate of MDM2. Hence, we determined whether HEY1 is subjected to MDM2-mediated degradation. When co-transfected into U2OS cells, ectopic expression of MDM2 dramatically reduced the steady-state level of wild-type HEY1, indicating that HEY1 might be targeted to proteasome-mediated degradation by MDM2. Moreover, simulation of HEY1 phosphorylation at residue S68 partially prevents MDM2-mediated HEY1 degradation (Figure 10F), in keeping with the idea that this phosphorylation event stabilizes HEY1 protein and reduces its ability to interact with MDM2. Nutlin-3 is a selective antagonist of MDM2 that binds MDM2 in the p53-binding pocket, thereby preventing the interaction between MDM2 and p53 and causing p53 stabilization ([32], Figure 10G, right panel). To investigate the possibility that HEY1 could also interact with MDM2 through the same p53-binding region we co-transfected MDM2 and HEY1 or p53 in the presence of Nutlin-3 and we observed that the drug was unable to protect HEY1 from MDM2-dependent degradation (Figure 10G, left panel). This result suggests that HEY1 would not compete with p53 for the binding to the same MDM2 region.

### Ribosomal stress causes HEY1 perinucleolar localization

**Figure 11:**
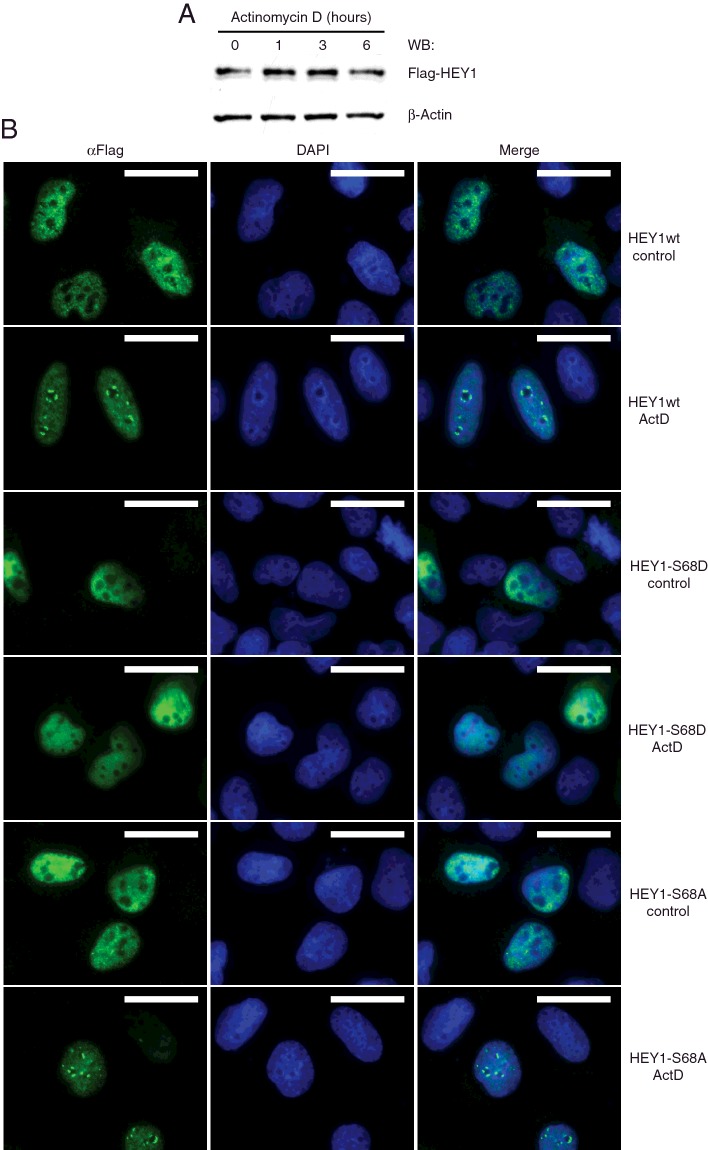
Ribosomal stress induced by actinomycin D causes HEY1 perinucleolar localization (**A**) U2OS cells previously transfected with expression vector for Flag-tagged HEY1 were treated with actinomycin D (5 nM) for 1, 3 or 6 h. HEY1 total protein levels were analysed by western blotting using anti-Flag antibody. (**B**) U2OS cells previously transfected with expression vectors for Flag-tagged HEY1, HEY1-S68D or HEY1-S68A were treated with actinomycin D (5 nM) for 6 h and assayed by indirect immunofluorescence with anti-Flag antibody. The first column shows the indirect immunofluorescence with anti-Flag antibody, the second column shows DAPI staining of DNA and the third column shows the merge image indicating the degree of colocalization. Bars, 20 μm.

To further explore a possible role for HEY1 in the modulation of p53 activation by ribosomal stress we studied the effects of treatment with the ribosomal stress-inducing agent actinomycin D on HEY1 total protein levels and cellular localization in U2OS osteosarcoma cells. U2OS cells were transfected with HEY1 expression vector and subsequently they were treated with 5 nM actinomycin D for 1 h, 3 h or 6 h. Western blot analysis showed that total wild-type HEY1 protein levels did not significantly change (Figure 11A). However, immunofluorescence analysis revealed that ribosomal stress causes a radical redistribution of HEY1 protein. In non-treated cells HEY1 presents uniform nuclear localization, however, 6 h after actinomycin D treatment HEY1 migrates towards the margin of the nucleoli (Figure 11B), accumulating in small structures, so-called nucleolar caps [33], formed by accumulation of nucleolar components during different stages of cellular metabolic activity. Based on differences in phase contrast light microscopy, two types of nucleolar caps have been described; dark nucleolar caps and light nucleolar caps [34]. We observed that HEY1 is found in phase light nucleolar caps (Supplementary Figure S5). Parallel experiments performed with HEY1 phosphorylation site mutants HEY1-S68A and HEY1-S68D revealed that, upon actinomycin D treatment, the non-phosphorylatable HEY1-S68A accumulated in the same nucleolar caps where wild-type HEY1 is found (Figure 11B). Strikingly, the mutant HEY1-S68D that simulates Ser-68 phosphorylation, which is defective for p53 activation and RPL11 interaction, does not change its uniform nuclear localization upon actinomycin D treatment (Figure 11B). These observations indicate that HEY1 could have previously unknown biological functions in the nucleolar stress pathway, and, again, these activities might be modulated by HEY1 phosphorylation at Ser-68. In accordance with these observations, in addition to the ribosomal proteins, 12 nucleolar proteins co-precipitated with HEY1 in our proteomic assay (Supplementary Table S5), reinforcing the notion about a potential role for HEY1 in the nucleolus during ribosomal stress. We confirmed that at least one of the nucleolar proteins, Non-Pou Domain Containing, Octamer-Binding (NONO), was able to interact with HEY1 *in vitro* by using a GST pull-down assay. Interestingly, this interaction is inhibited by simulation of HEY1 phosphorylation at residue S68 (Supplementary Figure S6), in keeping with the notion that this phosphorylation modulates HEY1 function during ribosomal stress. In view of the high conservation of the serine residue in the HLH domain of all bHLH-O proteins we examined the possible effects of ribosomal stress induced by actinomycin D in the subcellular distribution of HES1 and HEY2, two related members of the bHLH transcription family, also involved in the activation of the p53 pathway ([7], Figure 4A)). Thus, we observed that HES1 and HEY2 also accumulate in nucleolar caps in response to actinomycin D treatment (Supplementary Figure S7), suggesting that the bHLH-O transcription factors could share nucleolar functions.

## DISCUSSION

The p53 protein is a pleiotropic transcription factor that plays a crucial role in tumour suppression by regulating cell-cycle progression, apoptosis, senescence, angiogenesis and maintenance of genomic stability. The cellular responses to p53 activation depend on the type of stimuli, the duration of the signal, the cellular context and the cross-talk with other signal transduction pathways including TGF-β [35], Notch [36] and pRB [37], although the detailed molecular mechanisms responsible for the integration of these pathways are far from being fully elucidated. HEY1 could play a part in their cross-talk because activation of those pathways induces HEY1 expression [2–4], which then participates in the activation of p53. Also, we have observed that HEY1 can physically interact directly with p53 [20]. Therefore, alterations in HEY1 function and/or expression might contribute to oncogenesis. Here, we have uncovered a novel mechanism of regulation of HEY1 function and stability by phosphorylation at a conserved residue, Ser-68, located in the HLH domain. This serine is highly conserved among bHLH proteins and its phosphorylation could affect the ability of these proteins to form homo-and heterodimers and/or bind to DNA through the HLH domain. In keeping with this, our study revealed that simulation of HEY1 Ser-68 phosphorylation disturbs its ability to form heterodimers with HEY2, but not with HES1. This alteration in the binding specificity might represent an additional mechanism of control of bHLH-O protein dimer function. In addition, HEY1 interacts with the repressor complex Sin3 through its bHLH [38]), thus, HEY1 Ser-68 phosphorylation could also modulate HEY1 recruitment of transcriptional corepressors.

Simulation of HEY1 Ser-68 phosphorylation inhibits both its ability to enhance p53 transcriptional activity and its physical interaction with p53. We had previously shown that HEY1 protein is excluded from the nucleus in most human prostate cancers analysed [10], an alteration that would eliminate an activation signal for p53 tumour suppressor action [9]. However, the inability of HEY1-S68D phosphomimetic mutant to stimulate p53-dependent transcription does not reflect HEY1 exclusion from the nucleus, since the mutant retains nuclear localization. Interestingly, detection of phosphorylation *in vivo* by immunofluorescence with an anti-phosho-Ser-68-specific antibody revealed that there is accumulation of HEY1 phosphorylated at Ser-68 in the centrosome during mitosis, although we cannot exclude the possibility that phosphorylation of HEY1 Ser-68 occurs in other nuclear compartments at levels below the affinity range of our anti-phosho-Ser-68-specific antibody. Consistent with the observed centrosome localization we found numerous centrosome-associated proteins that co-immunoprecipitated with HEY1 in the proteomic study including centriolar structural proteins such as tubulins, Filamin-A, actin and Vimentin but also key regulators of pericentriolar material recruitment like CEP192 (Centrosomal Protein 192 kDa). Cell cycle-dependent accumulation of HEY1 phosphorylated at Ser-68 at centrosomes suggests that HEY1 has previously unknown functions in this organelle, that might be regulated via direct HEY1 phosphorylation. The pleiotropic functions of p53 as a cell cycle control protein include the regulation of centrosome homoeostasis and centrosome duplication (reviewed in [39]). Interestingly, just like in the case of phosphorylated HEY1, in mitosis, p53 localizes at the centrosomes in an ATM-dependent manner, where it has been proposed that monitors mitotic spindle integrity [40–42]. HEY1 could contribute to the modulation of p53 function in the centrosome although further studies will be required to investigate the possible role of HEY1, and other members of the bHLH-O family, in the centrosome. In this work we have identified two serine/threonine protein kinases that co-immunoprecipitated with HEY1 and can phosphorylate HEY1 Ser-68 *in vitro*, STK38 and STK38L, that belong to a subfamily of the AGC group of serine/threonine kinases highly conserved from yeast to man. Both kinases are part of an extended Hippo tumour suppressor pathway but they have also Hippo-independent cell cycle related functions (reviewed in [43]). Notably, although endogenous STK38 and STK38L are present in the nucleus and cytoplasm, a subpopulation of these kinases associates with centrosomes and is required for proper centrosome duplication [44]. Therefore, it is not surprising that substrates of these kinases are also localized in the centrosome. Our initial observations suggest that HEY1 could be a physiological substrate for STK38 kinases and open up the possibility that modulation of the activity of HEY proteins via direct phosphorylation may be one of the molecular mechanisms by which STK38 kinases exert their function. Centrosomes have recently become important as platforms for the integration of numerous signalling pathways (reviewed in [45,46]). Thus, HEY1, a downstream effector for several key transduction pathways, could affect centrosome-dependent functions, including cell cycle control, development and DNA damage response.

STK38 and STK38L promote cell cycle progression at different levels: increasing the activity of the proto-oncogene C-Myc [43,47], decreasing the stability of the cyclin-Cdk inhibitor protein p21 by direct phosphorylation at Ser-146 [48] and phosphorylating Heterochromatin protein 1α (HP1α) during mitosis [49]. In addition, cyclin D1 promotes cell cycle progression through enhancing STK38 kinase activity [50]. Since expression of wild-type HEY1, but not HEY1-S68D, causes p53-dependent cell cycle arrest in U2OS cells, inhibition of HEY1 ability to activate p53 transcriptional activity via direct phosphorylation at Ser-68 by STK38 kinases would integrate with the mitogenic signalling pathways induced by STK38 and STK38L.

We previously showed that expression of HEY1 sensitizes U2OS cells to p53-activating chemotherapeutic drugs, such us doxorubicin and cisplatin [9]. Here, we demonstrate that HEY1 expression also increases the sensitivity of U2OS cells to resveratrol, a dietary polyphenol with potential chancer chemopreventive properties (reviewed in [22]). However, expression of the mutant HEY1-S68D failed to increase cell sensitivity to any of the anticancer drugs tested, reinforcing the relevance of this regulatory phosphorylation event in the biological function of HEY1. Future work will determine the possible contribution of p53-dependent and p53-independent mechanisms on HEY1-mediated sensitization to chemotherapeutic drugs.

It was initially proposed that HEY1 activates p53 through repression of *MDM2* transcription, however HEY1 failed to bind to any identified potential binding sites in the *MDM2* promoter [7]. In addition, we have observed that HEY1 can interact directly with p53 [20]. Taken together, these observations suggest that there are mechanisms for the activation of p53 by HEY1 other than the transcriptional repression of *MDM2*. We found that HEY1 may have a stabilizing effect on p53 protein by binding to and inhibiting MDM2 E3 ubiquitin ligase activity, in a similar manner than the nucleolar protein ARF (alternative reading frame) or a number of ribosomal proteins that are released from the nucleolus in response to nucleolar stress. Our proteomic analysis revealed that several ribosomal proteins co-immunoprecipitate with HEY1, including seven of the ribosomal proteins shown to activate p53 by inhibiting MDM2 function, one of which is RPL11, an essential player during p53 induction in response to ribosomal stress with increasing relevance in tumour suppression [31,51,52]. We have confirmed that HEY1 interacts directly with RPL11 and, moreover, that both proteins cooperate in the inhibition of MDM2-mediated p53 degradation resulting in a synergistic positive effect on p53 transcriptional activity. These findings suggest that HEY1 could play a role in regulating p53 response to perturbations in ribosome biogenesis. In addition to the apparent inhibition of MDM2 activity caused by HEY1 expression, our results suggest that HEY1 could also be a physiological substrate of MDM2 since MDM2 down-regulates HEY1 protein levels. Interestingly, Nutlin-3 does not prevent MDM2-mediated HEY1 degradation, suggesting that the p53-binding pocket is not the main binding site for HEY1 and raising the possibility that HEY1, p53 and MDM2 could form ternary complexes. The elucidation of the intricate interplay between HEY1, MDM2, ribosomal proteins and p53 will require the identifications of the protein complexes formed *in vivo* and fine mapping of the domains responsible for the protein–protein interactions. Simulation of HEY1 Ser-68 phosphorylation prevents HEY1 interaction with MDM2 and RPL11. In keeping with this, the phosphomimetic mutant HEY1-S68D cannot protect p53 from MDM2-mediated degradation. The mutant HEY1-S68D also failed to migrate to nucleolar caps in response to ribosomal stress, also showing reduced interaction with the nucleolar protein NONO. Therefore, this functional interaction between HEY1 and the ribosomal protein-MDM2 axis seems to be regulated by its phosphorylation at Ser-68.

Taken together, our results suggest that signalling pathways that modulate HEY1 phosphorylation at Ser-68 could impinge on the activation of p53 tumour suppressor protein and also influence cellular sensitivity to chemotherapeutic drugs. Therefore, it may be potentially relevant to screen for alterations in the normal HEY1 phosphorylation levels at Ser-68 in human tumour samples. If an association between HEY1 phosphorylation status and the cancer phenotype can be found, STK38, STK38L and other serine/threonine kinases responsible for the phosphorylation of HEY1 at Ser-68 may represent novel attractive targets for therapeutic intervention. An important challenge for the future will therefore be the elucidation of the complex signalling network that connects HEY1 and these p53-activating pathways.

## Abbreviations

bHLH-O: basic helix–loop–helix-orange
HES1: hairy and enhancer of split 1
HEY1: hairy/enhancer-of-split related with YRPW motif 1
HEY2: hairy/enhancer-of-split related with YRPW motif 2
HEYL: hairy/enhancer-of-split related with YRPW motif like
MTS: 3-(4,5-dimethylthiazol-2-yl)-5-(3-carboxymethoxyphenyl)-2-(4-sulfophenyl)-2H-tetrazolium
pRB: retinoblastoma
STK38: serine/threonine kinase 38
STK38L: serine/threonine kinase 38 like
TGF-β: transforming growth factor β
